# Effects of Cyhalothrin-Based Pesticide on Early Life Stages of Common Carp (*Cyprinus carpio* L.)

**DOI:** 10.1155/2014/107373

**Published:** 2014-04-22

**Authors:** Zuzana Richterová, Jana Máchová, Alžběta Stará, Jitka Tumová, Josef Velíšek, Marie Ševčíková, Zdeňka Svobodová

**Affiliations:** ^1^Faculty of Fisheries and Protection of Waters, South Bohemian Research Center of Aquaculture and Biodiversity of Hydrocenoses, Research Institute of Fish Culture and Hydrobiology, University of South Bohemia in Ceske Budejovice, Zatisi 728/II, 389 25 Vodnany, Czech Republic; ^2^Department of Veterinary Public Health and Toxicology, Faculty of Veterinary Hygiene and Ecology, University of Veterinary and Pharmaceutical Sciences Brno, Palackeho trida 1/3, 612 42 Brno, Czech Republic

## Abstract

The effects of Nexide (a.i. gamma-cyhalothrin 60 g L^−1^) on cumulative mortality, growth indices, and ontogenetic development of embryos and larvae of common carp (*Cyprinus carpio* L.) were studied. Levels of oxidative stress parameters glutathione reductase (GR), glutathione peroxidase (GPx), catalase (CAT), glutathione-S-transferase (GST), and lipid peroxidation were determined. Eggs of newly fertilised common carp were exposed to Nexide at concentrations 5, 25, 50, 100, and 250 **μ**g L^−1^ (0.3, 1.5, 3, 6, and 15 **μ**g L^−1^ gamma-cyhalothrin). All organisms exposed to concentrations higher than 50 **μ**g L^−1^ died soon after hatching; at 25 **μ**g L^−1^, 95% mortality was recorded. Larvae exposed to 5 **μ**g L^−1^ showed significantly lower growth and retarded ontogenetic development compared to control. Histological examination of the livers of larvae from the exposed group revealed dystrophic changes. The value of detoxification enzyme GST of organisms from the exposed group was significantly higher compared to the control and the value of defensive enzyme GPx was significantly lower compared to the control. The results of our investigation confirmed that contamination of aquatic environment by pesticides containing cyhalothrin may impair growth and development of early life stages of carp and cause disbalance of defensive enzymes.

## 1. Introduction

Pyrethroids are synthetic analogues of natural pyrethrins that occur in the daisy (*Chrysanthemum cinerariaefolium*) and related species. Pyrethroids have replaced natural pyrethrins as agricultural pesticides, primarily due to their greater photostability. They act through disruption of the insect nervous system, leading to hyperactivity, paralysis, and death. Pyrethroids are among the most commonly used pesticides worldwide and pose a threat to the natural environment including nontarget organisms, such as fish that are highly sensitive. Contamination of surface water by pesticides is widespread [1, 2] and median concentrations lethal to fish of the more commonly used pyrethroids are generally less than 10 *μ*g L^−1^ [3].

Cyhalothrin is a pyrethroid that contains a cyano-3-phenoxybenzyl group. It blocks sodium channels of nerve filaments by lengthening their depolarization phase as well as affecting gamma-aminobutyric acid receptors that involve chloride and calcium channels in nerve filaments [4–7]. The presence of halogens in a formulation contributes to greater persistence and provides better residual activity against insects together with higher potential for negative effects on the environment [3].

Gamma-cyhalothrin is an insecticidal enantiomer of the synthetic pyrethroid lambda-cyhalothrin. Lambda-cyhalothrin consists of two of the four enantiomers of the cyhalothrin molecule. Different enantiomers of lambda-cyhalothrin show different toxicity to zebrafish (*Danio rerio*). The 24 h LC50 (−) enantiomer was reported as 2.03 *μ*g L^−1^, while 24 h LC50 of (+) enantiomer was >1.2 × 10^-2^ *μ*g L^−1^ [8]. Lambda-cyhalothrin consists of approximately 50% gamma-cyhalothrin, and its biological activity against pests and ecotoxicity to aquatic communities is relevant to this ratio [9].

The aim of this study was to evaluate the effect of Nexide with its active ingredient gamma-cyhalothrin on early life stages of common carp (*Cyprinus carpio* L.) using an embryo-larval toxicity test.

## 2. Materials and Methods

### 2.1. Experimental Substances

Cyhalothrin (S)-*α*-cyano-3-phenoxybenzyl(1R,3R)-3-[(Z)-2-chlor-3,3,3-trifluoropropenyl]-2,2-dimethylcyclopropanecarboxylate was tested in the form of the commercial preparation Nexide (Cheminova A/S, Denmark). Gamma-cyhalothrin as an active ingredient of this preparation was developed by Pytech Chemicals GmbH. The insecticide is a suspension of microcapsules containing 60 g L^−1^ gamma-cyhalothrin in an aromatic solvent intended for dilution in water.

### 2.2. Experimental Animals

Fertilised eggs of common carp were obtained from the breeding station of the Department of Fish Genetics and Breeding of the Research Institute of Fish Culture and Hydrobiology in Vodnany, Faculty of Fisheries and Protection of Waters, University of South Bohemia in Ceske Budejovice, Czech Republic. Eggs were produced according to standard methods of artificial reproduction [10].

### 2.3. Experimental Design

#### 2.3.1. Early Life Stage Toxicity Test

The test was based on the methodology of the OECD 210 Guideline for Testing of Chemicals [11]. The method was modified, in that only fertilised eggs were selected for testing. One hundred fertilised eggs were inserted to each crystallisation dish at 24 h after fertilisation. The ingested volume of control and each Nexide concentration was 1 L, and tested concentrations were 5, 25, 50, 100, and 250 *μ*g L^−1^ (0.3, 1.5, 3, 6, and 15 *μ*g L^−1^ gamma-cyhalothrin). The trial was performed in duplicate. Dechlorinated tap water (pH 7.98, N-NH_4_^+^ < 0.02 mg L^−1^, NO_2_^−^-N 0.006 mg L^−1^, NO_3_^−^-N 1.55 mg L^−1^, PO_4_^3−^-P 0.09 mg L^−1^, COD_Mn_/chemical oxygen demand with oxidizing agent potassium permanganate/0.6 mg L^−1^, and oxygen saturation > 80%) was used for dilution of test concentrations and for control baths. Daily monitoring was conducted to maintain temperature at 21–23°C, pH 7.5–8.5, and dissolved oxygen >60%. Each dish was continuously gently aerated, and the water bath was renewed once a day. Observations of hatching, survival, anatomy, and behaviour were made daily. Unhatched eggs and dead larvae were removed. Beginning day 8, larvae were fed by freshly hatched brine shrimp (*Artemia salina*) nauplii* ad libitum*. The beginning of the test was considered to be one day after fertilization and was designated day 1. Hatching was mainly completed on day 4, and feeding with* A. salina* was initiated on day 8. The test was concluded on day 35 when the majority of the fish in the control dishes reached the juvenile stage. Samples for observations of ontogenetic development, malformations, total length, and weight were taken on days 5, 12, 19, 26, 33, and 35. Ten samples were taken from the control and from the lowest Nexide concentration. At higher concentrations, this number was lower as a result of higher mortality of organisms. These samples were fixed in 4% formalin and examined after completion of the trial. Developmental stages comprised nine embryonic (E1–E9), six larval (L1–L6), and two juvenile (J1-J2) stages [12]. At the end of the test, samples of control and from the 5 *μ*g L^−1^ concentration were taken for histological examination. These samples were fixed in 10% formalin and processed using conventional paraffin techniques. Tissue sections were stained with haematoxylin and eosin. Slides of liver, intestine, kidney, and gill were examined at a magnification range 100–1000x by light microscopy.

#### 2.3.2. Cumulative Mortality and Biometric Data

Cumulative mortality was recorded daily, and samples of embryos/larvae were measured and weighed. Weight to the nearest 0.1 mg was measured by an analytical balance, WAS 220/C/2. Total length of embryos/larvae was measured using a binocular loupe and a scale to the nearest 0.01 mm. Fulton's condition factor (FCF) of fish surviving at the end of the trial was calculated using the formula FCF = *W*  × TL^−3^  × 100, where *W* is weight in g and TL is total length in cm.

#### 2.3.3. Determination of Oxidative Stress

Ten control fish and ten fish exposed to 5 *μ*g L^−1^ for investigation of oxidative stress were taken on day 35 and placed immediately into liquid nitrogen for transport to a screening laboratory. Whole bodies were homogenized in a 50 mM potassium phosphate buffer with 1 mM EDTA (pH 7.4) and centrifuged at 11,000 g for 20 min at 4°C. The supernatant was pipetted into individual Eppendorf tubes and kept at −85°C until analysis. Supernatant was used for determination of glutathione reductase (GR), glutathione peroxidase (GPx), catalase (CAT), glutathione-S-transferase (GST), and protein concentration. Noncentrifuged homogenate (stored at −85°C) was used to estimate lipid peroxidation. Protein concentration was quantified with the Bicinchoninic Acid Protein Assay Kit (Sigma-Aldrich, St. Louis, MO, USA) using bovine serum albumin as a standard [13]. Total catalytic concentration of GST was determined by measuring the conjugation of 1-chloro-2,4-dinitrobenzene with reduced glutathione at 340 nm [14]. Specific activity was expressed as the nmol of the formed product per min per mg of protein. The catalytic concentration of GR was determined by measuring NADPH oxidation at 340 nm [15]. The catalytic concentration of GPx was calculated from the rate of NADPH oxidation by the reaction with GR at 340 nm [16]. Specific activity of GR and GPx was expressed as nmol of NADPH consumption per min per mg of protein. The activity of CAT was determined by measuring H_2_O_2_ breakdown at 240 nm and expressed as *μ*mol of decomposed H_2_O_2_ per min per mg of protein [17]. Lipid peroxidation was determined using the thiobarbituric acid-reactive substances (TBARS) method at 535 nm [18]. The concentration was expressed as nmol per g wet weight of tissue. All parameters were measured spectrophotometrically using a Varioskan Flash spectral scanning multimode reader (Thermo Fisher Scientific Inc., Waltham, MA, USA).

#### 2.3.4. Determination of the Active Ingredient

Cyhalothrin in water samples was determined after extraction using isooctane by gas chromatography with electron capture detection (GC/ECD) [19]. Chromatography was performed on a column HP-5MS (60 m × 0.32 mm, 0.25 *μ*m). The carrier gas was helium with a flow rate of 25 mL min^−1^ and a splitless injection volume of 2 *μ*L and temperature of 250°C was used. Temperature column program was 100°C for 2 min, increased to 230°C at 14°C min^−1^, increased to 285°C at 4°C min^−1^, and held at 285°C for 40 min. This method was used to confirm the presence of cyhalothrin, the active substance at >80% throughout the test.

#### 2.3.5. Statistical Analyses

The software program Statistica, v. 10.0, for Windows (StatSoft, Prague, Czech Republic) was used to compare differences among the test groups. Prior to analysis, all measured variables were checked for normality (Kolmogorov-Smirnov test) and homoscedasticity of variance (Bartlett's test). If those conditions were satisfied, a one-way ANOVA was employed to determine whether there were significant differences in measured variables among experimental groups. When a difference was detected (*P* < 0.05), Dunnett's multiple-range test was applied. If the conditions for ANOVA were not satisfied, a nonparametric test (Kruskal-Wallis) was used. Normality of oxidative stress data was assessed by Shapiro-Wilk test; data were normally distributed. Test of homogeneity of variance (Levene's test) and an analysis of variance (ANOVA) test were performed, followed by multiple comparisons (Tukey-HSD test). Differences were considered to be significant when *P* < 0.05.

## 3. Results

### 3.1. Cumulative Mortality

**Figure 1:**
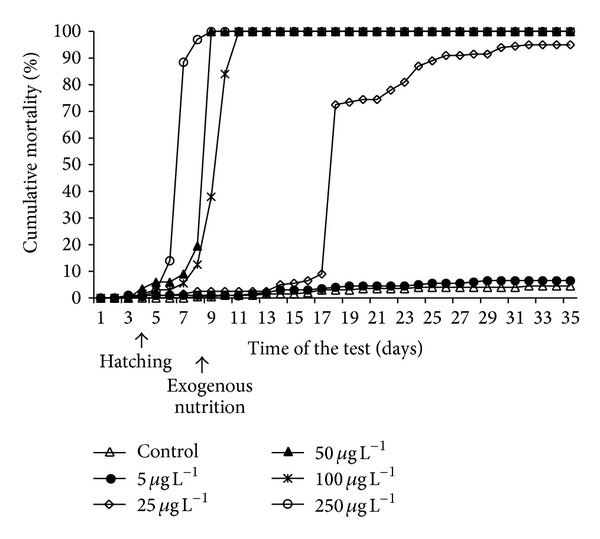
Cumulative mortality of common carp embryos and larvae during embryo-larval toxicity test with Nexide.

Only small differences in mortality were observed among test groups and control days 1–5 (Figure 1). The first mortalities appeared on day 3 in 5, 25, and 250 *μ*g L^−1^ concentrations. On day 6 delayed and reduced hatching along with posthatch mortality was observed with 1%, 1%, 6%, 3%, and 14% mortality at 5, 25, 50, 100, and 250 *μ*g L^−1^, respectively. No mortality occurred in the control group. Surviving embryos/larvae in the 250 *μ*g L^−1^ exposure showed almost no movement, while control and those exposed to lower concentrations swam normally. Total mortality was observed in 50 and 250 *μ*g L^−1^ concentrations on day 9 and in 100 *μ*g L^−1^ on day 11. Day 18 of the test was accompanied by heavy mortality in the 25 *μ*g L^−1^ group. At the conclusion of the trial, 93.5% larvae were viable in the 5 *μ*g L^−1^ group and 5% in the 25 *μ*g L^−1^ concentration, compared to a 95.5% survival rate in the control group.

### 3.2. Length and Weight Growth

**Figure 2:**
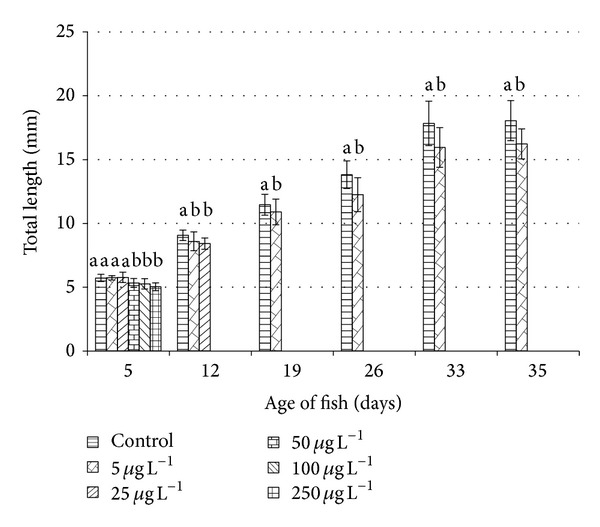
Effect of Nexide on total length (mean ± SD) of common carp larvae and juveniles during embryo-larval test. Significant differences (*P* < 0.05 on days 5–26 and *P* < 0.01 on days 33 and 35) between groups at each sampling time are indicated by different letters (a, b).

**Figure 3:**
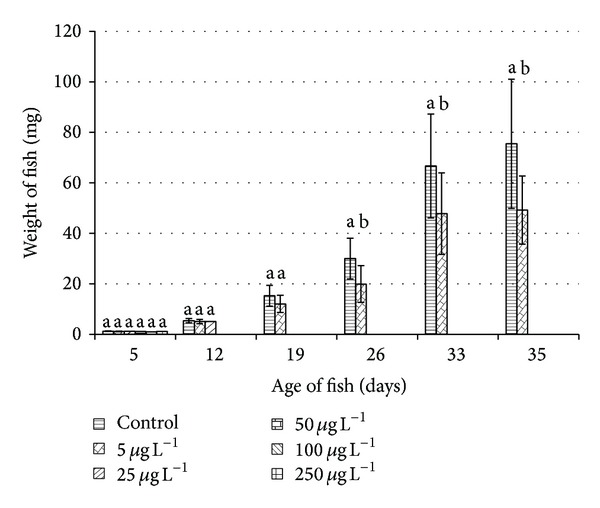
Effect of Nexide on weight (mean ± SD) of common carp larvae and juveniles during embryo-larval test. Significant differences (*P* < 0.05 except day 26 when *P* < 0.01) between groups at each sampling time are indicated by different letters (a, b).

Growth in total length and weight and ontogenetic developmental stage are recorded (Figures 2 and 3). Samples from all concentrations were taken only on day 5, because no larvae survived beyond day 11 in 50, 100, and 250 *μ*g L^−1^ exposure. Only the control and the 5 *μ*g L^−1^ group were compared at the completion of the trial. Nineteen larvae/juvenile fish from the control group and ten larvae exposed to 5 *μ*g L^−1^ were examined on day 35. Insufficient individuals survived in the 25 *μ*g L^−1^ group to be included in growth comparisons.

FCF was calculated as an index of thriving in fish from the control group and the 5 *μ*g L^−1^ concentration on day 35. The difference between control (1.174 ± 0.054) and concentration of the 5 *μ*g L^−1^ (1.131 ± 0.085) was significant (*P* < 0.05).

### 3.3. Early Ontogeny

From day 26, developmental stages of control and the 5 *μ*g L^−1^ group showed differences. No fish from the 5 *μ*g L^−1^ concentration reached the juvenile stage by the end of the test, remaining at larval stage 6 (Table 1). Macroscopic morphological anomalies such as curvature of the spine, changes in yolk sac, and shortening of body were rare in both groups and could be considered chance occurrences. A higher incidence of deeper pigmentation was observed in fish from the 5 *μ*g L^−1^ concentration.

### 3.4. Histopathology

**Figure 4:**
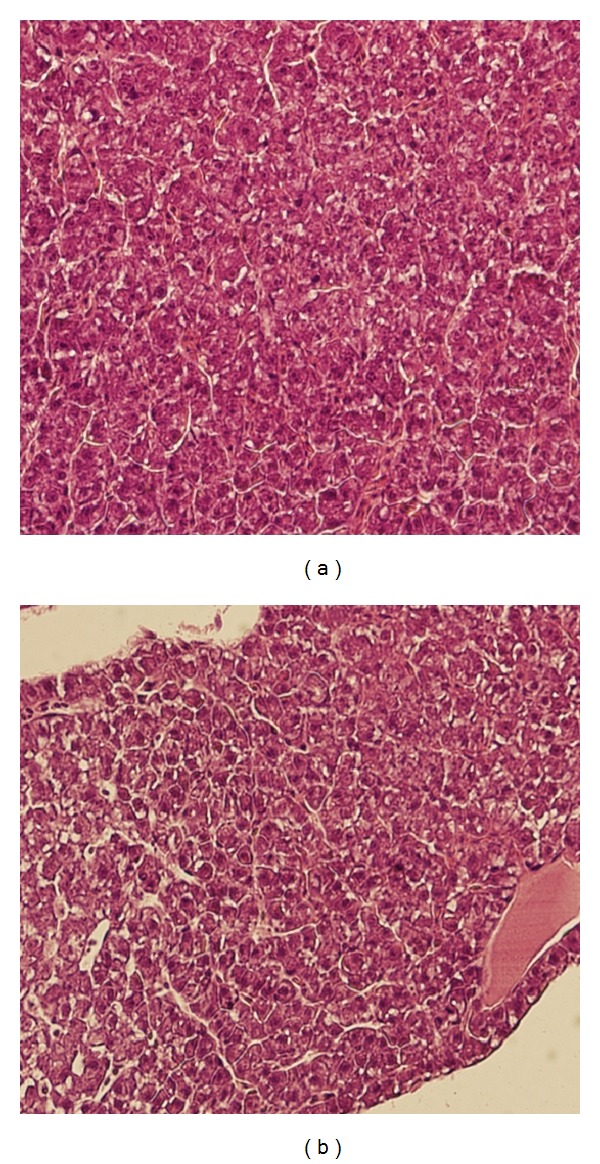
Liver of common carp larvae and juveniles on day 35 of embryo-larval toxicity test (400x). Control group (a) and 5 *μ*g L^−1^ Nexide (b) (photo by F. Tichý).

**Figure 5:**
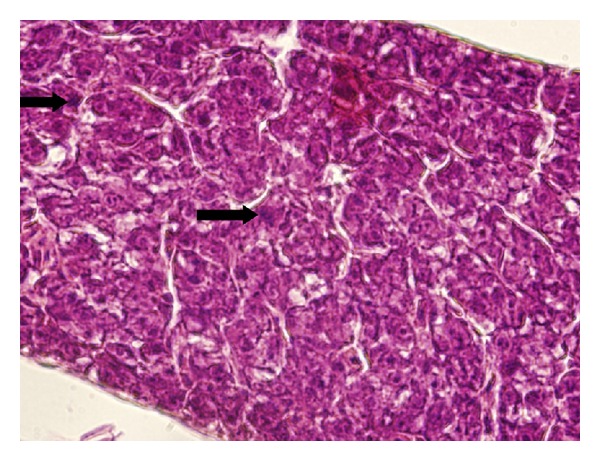
Rare mitotic structures (arrows) in liver of common carp larvae and juveniles exposed to 5 *μ*g L^−1^ Nexide on day 35 of embryo-larval test (1000x) (photo by F. Tichý).

Light microscopy revealed significant differences in steatosis dystrophy in liver of fish exposed to Nexide at concentration of 5 *μ*g L^−1^. The extent and degree of hepatodystrophic changes appeared clearly at magnitude 400x (Figure 4). Magnification to 1000x revealed rare mitotic structures in liver cells of fish exposed to Nexide at concentration of 5 *μ*g L^−1^ (Figure 5). The intestine, kidney, and gill of 5 *μ*g L^−1^ and control did not show significant differences.

### 3.5. Effect on Oxidative Stress

**Figure 6:**
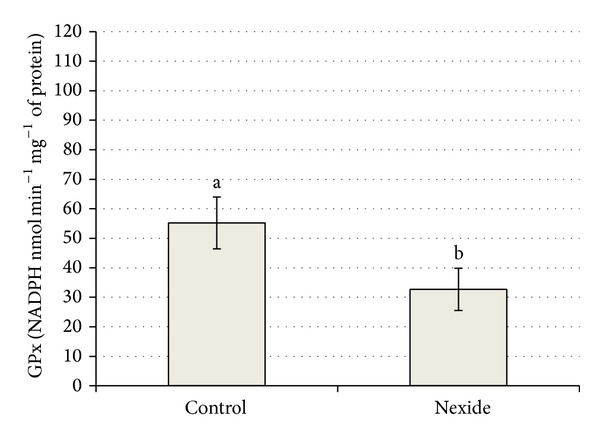
Effect of Nexide at 5 *μ*g L^−1^ on GPx level in common carp larvae and juveniles on day 35 compared to control (*P* < 0.01).

**Figure 7:**
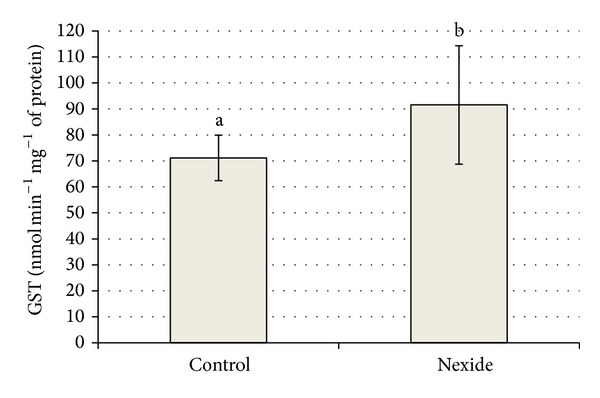
Effect of Nexide at 5 *μ*g L^−1^ on GST level in common carp larvae and juveniles on day 35 compared to control (*P* < 0.05).

Levels of oxidative stress parameters of control and the 5 *μ*g L^−1^ concentration on day 35 are summarized in Figures 6 and 7. A significant decrease (*P* < 0.01) of GPx and significant increase (*P* < 0.05) of GST in 5 *μ*g L^−1^ group compared to control are shown in Figures 6 and 7.

Mean level of GR in control was 9.8 ± 1.36 compared to 11.3 ± 2.36 nmol of NADPH min^−1^ mg^−1^ of protein in 5 *μ*g L^−1^ group. Mean level of TBARS in control and in 5 *μ*g L^−1^ group was 12.1 ± 2.58 and 10.1 ± 4.19 nmol g^−1^ wet weight, respectively, and mean level of CAT was 31.4 ± 5.07 and 27.7 ± 7.32 *μ*mol of H_2_O_2_ min^−1^ mg^−1^ of protein, respectively. There were no significant differences in GR, TBARS, and CAT levels between control and treated fish.

## 4. Discussion

The present study revealed significantly reduced growth, delayed and reduced hatching, and high mortality after hatching, especially at concentrations of 250 *μ*g L^−1^. Behaviour changes were observable as minimal movement of fish in 250 *μ*g L^−1^ preceding death. Histopathological examination revealed steatosis dystrophy in fish at the 5 *μ*g L^−1^ concentration. These findings are consistent with the toxic effects of common pyrethroids on fish [20]. Significantly lower FCF were observed in fish from concentration 5 *μ*g L^−1^ compared to control. Our results were in agreement with reports demonstrating a decline in condition factor in fish exposed to environmental pollutants [21], although this finding is not universal, as studies have reported no differences in common carp exposed to deltamethrin compared to control [22].

Exposure ≥50 *μ*g L^−1^ of Nexide resulted in death of all embryos/larvae. Lower concentrations did not cause 100% mortality but were associated with differences in biochemical parameters of oxidative stress and microscopic appearance of liver. The toxic effects of cyhalothrin described in literature are mainly associated with lambda-cyhalothrin, but the toxicity of isomers has been shown to differ. A comparison of 96 h LC50 values of gamma- versus lambda-cyhalothrin revealed respective levels of 0.047 *μ*g L^−1^ versus 0.149 *μ*g L^−1^ in bluegill (*Lepomis macrochirus*), 0.111 *μ*g L^−1^ versus 0.214 *μ*g L^−1^ in rainbow trout (*Oncorhynchus mykiss*), 0.17 *μ*g L^−1^ versus 2.3 *μ*g L^−1^ in guppy (*Poecilia reticulata*), and 0.27 *μ*g L^−1^ versus 0.64 *μ*g L^−1^ in zebrafish [9]. The 96 h LC50 lambda-cyhalothrin value for carp was reported at 0.5 *μ*g L^−1^ [23], and the 96 h LC50 lambda-cyhalothrin value for juvenile Nile tilapia (*Oreochromis niloticus*) was 2.901 *μ*g L^−1^ [24]. Thus we may presume that toxicity of lambda-cyhalothrin to early life stages of common carp may not be as significant as our results using gamma-cyhalothrin.

Oxidative stress parameters in whole body homogenates of larvae common carp in 5 *μ*g L^−1^ group revealed greater GST activity and lower GPx level compared to control, while differences in TBARS, GR, and CAT were not significant. Lambda-cyhalothrin was shown to lead to oxidative stress in liver of* O. niloticus *by increasing such indicators as lipid peroxidation, total glutathione (tGSH), GSH, TBARS content, and GST activity. An adaptive response was mounted by tGSH, GSH, and GSH-dependent enzymes. Oxidative stress is shown to upregulate GSH and GSH-related enzymes [24]. High levels of the antioxidant enzymes superoxide dismutase (SOD) and CAT followed exposure to cypermethrin in common carp. The enhanced lipid peroxidation in blood and tissue showed that cypermethrin-induced reactive oxygen species (ROS) were not completely scavenged by the antioxidant enzymes [25]. Many pesticides have been shown to be associated with production of oxidative stress in aquatic organisms, because they may induce the formation of ROS and alterations in antioxidant or free oxygen radicals scavenging enzyme systems [26–29]. Lambda-cyhalothrin has been reported to lead to oxidative stress by altering antioxidant systems and increasing lipid peroxidation in mammals [30, 31].

The present study did not show all pyrethroid effects previously reported, but influence on early developmental stages of common carp was clear. We found significant increase in mortality dependent on dose and duration of exposure. Acute toxicity tests of deltamethrin and cypermethrin on embryos and larvae of common carp have also shown dose-dependent decrease of hatching success [32, 33]. Our results agree that fish embryos appear to be less sensitive to pyrethroids than larvae [20].

## 5. Conclusion

Embryo-larval test on common carp with Nexide (containing 60 g L^−1^ of active substance gamma-cyhalothrin) revealed the following.

- Concentration of 250 *μ*g L^−1^ caused 100% mortality of embryos after hatching.
- Concentrations of 100 and 50 *μ*g L^−1^ caused 100% mortality soon after beginning of exogenous nutrition.
- Concentration of 25 *μ*g L^−1^ caused 95% mortality of exposed organisms during 35 days.
- The lowest tested concentration of Nexide (5 *μ*g L^−1^) caused slightly elevated mortality compared to the control group, significantly lower growth (length and weight), and retarded ontogenetic development. Also dystrophy in liver, significantly greater activity of detoxification enzyme GST, and lower levels of defensive enzyme GPx compared to control were observed.

The results of our investigation confirmed that contamination of aquatic environment by pesticides containing cyhalothrin may impair growth and development of early life stages of carp and cause disbalance of defensive enzymes. That is why we recommend further attention to studies of long-term effects of pyrethroids on fish and focus the investigation on offspring quality and their susceptibility to infectious diseases.

## Figures and Tables

**Table 1:** Ontogeny of common carp exposed to Nexide at 5 µg L^−1^ compared to control.

Sampling day	Developmental stages	
	Control	5 µg L^−1^
Day 5	E8-E9	E8-E9
Day 12	L3-L4	L3-L4
Day 19	L4-L5	L4-L5
Day 26	L5-L6	L5
Day 33	L6-J1	L6
Day 35	L6-J1	L6
